# Insomnia, Health-Related Quality of Life and Health Outcomes in Children: A Seven Year Longitudinal Cohort

**DOI:** 10.1038/srep27921

**Published:** 2016-06-13

**Authors:** Daniel Combs, James L. Goodwin, Stuart F. Quan, Wayne J. Morgan, Safal Shetty, Sairam Parthasarathy

**Affiliations:** 1 Department of Pediatrics, University of Arizona, Tucson, AZ, USA; 2 Department of Medicine, University of Arizona, Tucson, AZ, USA; 3 Arizona Respiratory Center, University of Arizona, Tucson, AZ, USA; 4 Division of Sleep and Circadian Disorders, Brigham and Women’s Hospital and Division of Sleep Medicine, Harvard Medical School, Boston, MA, USA; 5 Center for Sleep Disorders and Division of Pulmonary, Allergy, Critical Care and Sleep Medicine, University of Arizona, Tucson, AZ, USA.

## Abstract

Insomnia is common in children, and is associated with decreased school performance and increased psychopathology. Although adult insomnia is linked to worsened health-related quality of life (HRQOL), there is insufficient data evaluating insomnia and HRQOL in children. We examined the HRQOL and health associations of insomnia in a longitudinal cohort of 194 children (96 girls, age at study start 8.7 ± 1.6 years, age at data analysis 15.0 ± 1.8 years) over 7 years. International Classification of Sleep Disorders, second edition (ICSD2) derived insomnia was seen intermittently in 27% of children, and was persistent in 4%. Children reporting ICSD2-derived insomnia had lower HRQOL. Additionally, the presence of insomnia was associated with an increased risk of reporting a new medical condition (intermittent insomnia odds ratio 5.9 [95% CI 1.3–26.7, p = 0.04], persistent insomnia odds ratio 8 [95% CI 2.3–27.7, p = 0.001]). Persistent ICSD2-derived insomnia was associated with an increased risk of reporting a new medication (odds ratio 4.9 (95% CI 1.0–23.6), p = 0.049), and reporting a new psychiatric medication (odds ratio 13.7, 95% CI: 2.6–73.5, p = 0.002). These associations were present even after adjusting for socioeconomic factors and the presence of obstructive sleep apnea. Insomnia in children is associated with worsened HRQOL and health outcomes.

Insomnia is common in children. In preschool children (age 4), the prevalence is estimated to be 36%, while in elementary school children (age 5–10), the prevalence is estimated to be 20%^1^. The prevalence in adolescents (age 11–17) ranges from 4 to 23.8%^2^,^3^. There are limited longitudinal studies of insomnia in children, but data available show that persistence of insomnia symptoms ranges from 15 to 60%^4^,^5^ in school age children as they became adolescents, with a lower rate of persistence in infants and preschool children as they became school age, 0.3–21%^6^,^7^,^8^. These studies have used different diagnostic criteria, such as requirement of daytime symptoms or varied required frequency of symptoms; and some assessed sleep problems rather than insomnia^6^, which may explain the variance of the reported prevalence. A recent five year longitudinal study using a more strict criteria for insomnia showed persistence of insomnia in 14.9% of adolescents for insomnia occurring ≥3 times per week, and 27% using a less strict insomnia definition with symptoms occurring only once per week^5^.

Insomnia has been associated with multiple negative outcomes in adults and children. Insomnia in adults has been linked to decreased health-related quality of life (HRQOL)^9^,^10^ and mental health problems^11^. Additionally, persistent insomnia in adults has been linked to increased inflammation, mortality^12^,^13^ and increased likelihood of reporting a chronic disease^14^. In elementary school children (age 5–10), poor sleep quality and insomnia are associated with decreased school performance. In adolescents (age 11–17), poor sleep quality and insomnia are associated with decreased school performance^15^,^16^, increased psychopathology^17^ and increased risk of self-harm and suicidal ideation^18^,^19^. Chronic insomnia has specifically been associated with increased risk of somatic health problems, interpersonal problems, psychological problems, and problems with daily activities in adolescents^20^,^21^. Similarly, chronic insomnia has been associated with psychological problems in school-age children^1^. The presence of persistent insomnia in school age children has been associated with increased mental health symptoms at age 18^1^.

Although insomnia is linked to worsened HRQOL in adults^9^ and difficulty falling asleep and nocturnal awakenings have been shown to impair HRQOL in preschool age children^22^, the impact of insomnia on HRQOL in school age children has not been previously reported. We performed a secondary analysis of the Tucson Children’s Assessment of Sleep Apnea (TuCASA) study to determine the effects of intermittent and persistent insomnia on HRQOL in children. In addition, given the known associations between insomnia and chronic disease^14^, as well as insomnia and mental health problems^17^,^18^,^20^, we evaluated the association between the presence of intermittent (present at only one time point, or resolved by the final time point) or persistent insomnia (present in at least the last two time points), and the odds of reporting a new chronic medical condition or new medication. We evaluated 2 models of insomnia, one requiring nighttime symptoms alone, and the other derived from the 2nd edition of the International Classification of Sleep Disorders (ICSD2)^23^. In the ICSD2, insomnia is defined by a complaint of difficulty initiating sleep, difficulty maintaining sleep, or waking up too early; or sleep that is chronically unrestorative or poor in quality; which occurs despite adequate opportunity and circumstances for sleep. Additionally, at least one form of daytime impairment related to the nighttime sleep difficulty is reported: fatigue or malaise; attention, concentration, or memory impairment; social or vocational dysfunction or poor school performance; mood disturbance or irritability; daytime sleepiness; motivation, energy, or initiative reduction; proneness for errors or accidents at work or while driving; tension, headaches, or gastrointestinal symptoms in response to sleep loss; or concerns or worries about sleep.

## Methods

**Figure 1:**
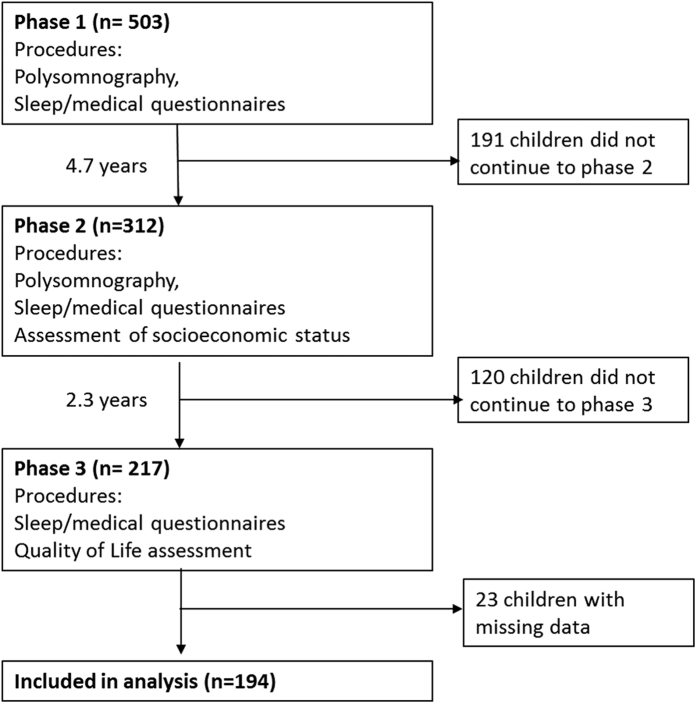
Flow chart of study procedures and attrition.

We performed a secondary analysis of the data derived from phases 1, 2 and 3 of the TuCASA community-based longitudinal cohort study. A detailed description of the TuCASA study and recruitment has been reported elsewhere^24^,^25^. In brief, a representative sample of 503 elementary school aged Caucasian and Hispanic children were recruited from the Tucson Unified School District, a large district representative of the Tucson population. Approximately 5 years later (mean 4.7 years), 348 children participated in phase 2 of the study. Two years later (mean 7.5 years from phase 1 and 2.3 years from phase 2), 217 children participated in phase 3. The TuCASA study was approved by the University of Arizona Institutional Review Board and the Tucson Unified School District Research Committee, and all methods were carried out in accordance with the approved guidelines. Prior to undergoing study-related procedures, written informed consent and minor’s assent were obtained from the parents and child respectively. Detailed sleep questionnaires were filled out at all phases. At phase 1, these were filled out by parents, at phase 2, both parent and child provided responses, and at phase 3, self-report was obtained. A participant flow chart is shown in Fig. 1 and specific question details are reported in the supplemental information. For analysis, the child-reported symptoms were used at phase 2.

### Insomnia Criteria

The presence of insomnia was derived from the 2^nd^ edition of the International Classification of Sleep Disorders (ICSD2) criteria^23^. Specifically, symptoms of trouble falling asleep, staying asleep, or waking up too early in the morning, accompanied by at least one daytime symptom and adequate opportunity to sleep. Daytime symptoms included learning problems, daytime sleepiness, or concern about not getting enough sleep. The presence of only a single daytime symptom was considered sufficient to fulfill this criterion. To evaluate adequate opportunity for sleep, a time in bed duration was calculated from subject reported weekday bedtime to wake time at each phase. This methodology has been previously used in studies evaluating an ICSD2 based definition of insomnia as a measure of opportunity for sleep^26^. We used a minimum of 9 hours in bed, based on recent recommendations^27^,^28^,^29^. We evaluated two models for case-determination of insomnia. The first model required only the subjective report of nighttime symptoms, including trouble falling asleep, staying asleep, or waking up too early in the morning. The second model had more restrictive criteria derived from the ICSD2. This definition of insomnia included a subjective complaint of problems falling asleep, staying asleep or waking too early in the morning with the additional requirements of ≥9 hours of time in bed as well as at least one daytime symptom including learning problems, daytime sleepiness, or concern about not getting enough sleep. Sensitivity analysis was also performed using several thresholds for adequate time in bed. These included a minimum of 7 or 8 hours of time in bed, as well as an age-adjusted threshold requiring 9 hours for children and 8 hours for adolescents. Sensitivity analysis was also performed using parent-report compared to child-report at phase 2.

Based on reported insomnia, children were classified into three groups – never, intermittent or persistent insomnia. Children without reports of insomnia during any of the three phases were categorized as never insomnia. Children with insomnia reported at only one phase, phase 1 and phase 3 only, or phase 1 and 2 only were classified as having intermittent insomnia. Children with insomnia at phase 2 and 3, or at all phases were classified as persistent insomnia.

### Quality of Life Assessment

The teen self-report version of the Pediatric Quality of Life Inventory (PedsQL) was used to measure HRQOL in phase 3 only^30^. This includes 23 questions that evaluate four subscales: physical functioning, emotional functioning, social functioning and school functioning. A total score was computed from all four subscales and a psychosocial health summary score is computed from the emotional, social and school functioning subscales. Scores range from 0–100, with a higher score indicating better HRQOL.

### Obstructive Sleep Apnea

One night of unattended polysomnography was used in phase 1 and 2 to determine the presence of obstructive sleep apnea (OSA). Complete details of the polysomnography have been reported previously^24^,^31^. An apnea-hypopnea index of ≥1.5 was used as the threshold to determine the presence of OSA^32^. In phase 3, participants were asked if they had been diagnosed with obstructive sleep apnea. A self-report of yes was considered to be representative of OSA.

### Medical Data

Family-reported medical information was collected at all phases. Specifically, participants were asked if they were taking any medications, and if they had any medical conditions for which they were under care of a physician. If participants answered yes to either question, they were also asked to provide the specific medications or conditions. A child was considered to be taking a medication if they provided the name of any medication with the exception of supplements or contraceptive agents. A child was determined to be using a psychiatric medication if an anti-depressant, stimulant, sedative or anti-psychotic medication was listed. To determine if a child was taking new medication or had a new medical condition, the reported medical conditions and medications at phase 3 were compared to the reported medical conditions and medications at phase 1. The report of a medical condition/medication at phase 3 that was not given during phase 1 was used to define reporting a new medical condition/medication.

### Socioeconomic Data

Socioeconomic information was derived from the phase 2 questionnaire. This information included family reported household income, parental education and ethnicity. Household income was obtained on a scale ranging from less than $5,000 annual income to greater than $50,000 annual income, in $5,000 increments. For data analysis, household income was collapsed into a binary value of less than or greater than $40,000 annual income as done previously^33^. This threshold income was chosen because it is representative of the median annual household income in Tucson, AZ at the time of data collection ($38,800)^34^. Parental education (of the caregiver filling out the survey) was assessed using a scale starting at high school education or less to greater than 4 years of college or professional school. The scale was subdivided into one year increments for completion of technical/trade school or college. Parental education was converted into a binary value of less than or greater than 4 years of college.

### Data Analysis

Statistical analysis was performed using SPSS Version 23 (IBM, Armonk, NY). To assess for demographic differences between groups, Pearson χ2 tests or Fisher exact tests were used for categorical variables and one-way analysis of variance (ANOVA) was used for continuous variables. To assess for differences in HRQOL, analysis of covariance (ANCOVA) with Bonferroni correction for multiple comparisons was performed. Adjustment was performed for the presence of OSA, household income, parental education, ethnicity, age, gender and use of psychiatric medication. Use of psychiatric medication was included as an adjustment for HRQOL outcomes as the presence of a psychiatric condition^35^,^36^ and the use of psychiatric medications^37^,^38^ are associated with worsened HRQOL in children. Specific HRQOL measures included the PedsQL total score, psychosocial composite score, and physical, social, emotional and school subscales. For health outcomes analysis, binary logistic regression with adjustment for the presence of OSA, household income, parental education, ethnicity, age and gender was performed. Specific health measures examined included reporting a new medical condition, new medication, or new psychiatric medication at phase 3. A p-value of less than 0.05 was considered significant.

## Results

A total of 217 children participated in phases 1, 2 and 3 of the TuCASA study and 194 children had complete data for all variables and were included in data analysis. Comparison of the attrition sample compared to the study cohort showed that there was a significantly higher attrition among Hispanic children and children with a lower family income (Table 1). No difference was seen in the rate of insomnia or OSA at phase 1 between the participants who remained in the study versus participants who discontinued participation. Longitudinal demographic information is reported in Table 2. There was an even distribution of girls and boys, with a predominance of Caucasian compared to Hispanic children. Transient insomnia was common at all time points (Table 2).

Either intermittent or persistent insomnia defined by night-time symptoms alone was present in 147 children (76%). Intermittent insomnia was reported by 82 (42%), and 65 (34%) reported persistent insomnia. ICSD2-derived insomnia was reported by 59 children (30%), with 52 (27%) reporting intermittent insomnia and 7 (4%) reporting persistent insomnia. We also evaluated an alternative definition of ICSD2-derived insomnia, using an age adjusted time in bed of 9 hours at phase 1 and phase 2, but only 8 hours at phase 3, based on recent guidelines^27^. Under this definition, insomnia was reported by 76 children, with 63 (33%) reporting intermittent insomnia, and 13 (7%) reporting persistent insomnia.

Demographic information by frequency of reported insomnia is provided in Table 3. For insomnia determined by nighttime symptoms only, there were no significant differences in age, gender, race, parental education or household income between participants with never, intermittent or persistent insomnia. The use of psychiatric medications was significantly greater in participants with ICSD2-derived insomnia, and participants with persistent insomnia were younger than those with never insomnia or intermittent insomnia (Table 3).

Polysomnography results were evaluated at phase 1 and phase 2. The presence of insomnia (regardless of method of definition) at a phase was not associated with significant differences in sleep architecture at that phase. Specifically, there were no differences in sleep latency, time in bed, total sleep time, or sleep efficiency. There were also no differences in percentage of the night in stage N1, N2, N3 or REM sleep. Supplemental Table 3 provides further details of polysomnography results.

**Figure 2:**
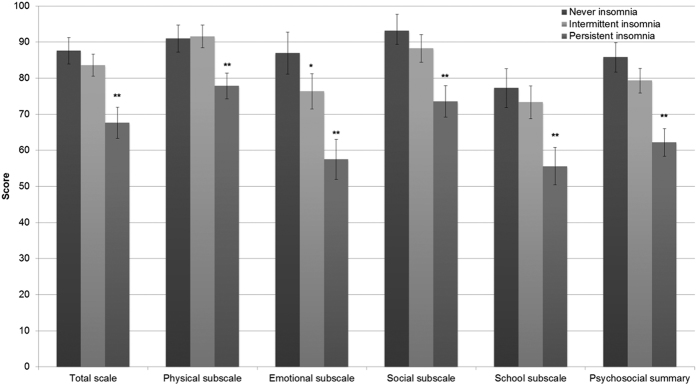
PedsQL Scores in participants with nocturnal symptom-based insomnia. The presence of insomnia is associated with decreased HRQOL across multiple domains of the PedsQL. This effect was greater in in the total, emotional, social and psychosocial scores of individuals with persistent insomnia. *Significantly different (p < 0.05) from no insomnia, **significantly different from intermittent insomnia. Insomnia was defined as presence of at least one of the following symptoms: trouble falling asleep, staying asleep, or waking up too early in the morning. PedsQL scores were adjusted for age, gender, ethnicity, family income, parent education, reported oat least one of the following daytime symptoms bstructive sleep apnea and use of psychiatric medication.

Intermittent and persistent insomnia–regardless of method of definition – were associated with worse HRQOL as measured by the PedsQL (Table 4). PedsQL scores, adjusted for age, gender, ethnicity, family income, parent education, OSA and use of psychiatric medication are shown in Fig. 2 for never, intermittent or persistent insomnia. In general, persistent in comparison to intermittent insomnia was associated with worse HRQOL across all PedsQL domains. Specifically, intermittent insomnia was associated with significantly worse HRQOL on the emotional domain. However, persistent insomnia was associated with significantly worse HRQOL compared to no insomnia as well as intermittent insomnia across all domains and the total scale (Fig. 2).

**Figure 3:**
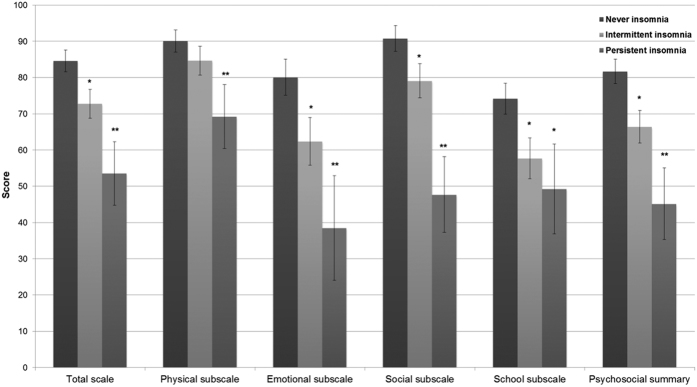
PedsQL Scores in participants with ICSD2-derived insomnia. The presence of insomnia is associated with decreased HRQOL across all domains except the physical scale. Persistent insomnia is associated with further worsening of overall, social and psychosocial HRQOL. *Significantly different (p < 0.05) from no insomnia, **significantly different from intermittent insomnia. Insomnia required the presence of at least one of the following symptoms of trouble falling asleep, staying asleep, or waking up too early in the morning in addition to reporting adequate time in bed (≥9 hours) and the presence of at least one of the following daytime symptoms–learning problems, daytime sleepiness, or concern about not getting enough sleep. PedsQL scores were adjusted for age, gender, ethnicity, family income, parent education, reported obstructive sleep apnea and use of psychiatric medication.

PedsQL scores, adjusted for age, gender, ethnicity, family income, parent education, reported obstructive sleep apnea and use of psychiatric medication by ICSD2-derived insomnia category are shown in Fig. 3. ICSD2-derived intermittent insomnia was associated with significantly lower HRQOL compared to never insomnia in the total score, emotional, social, school and psychosocial scales. ICSD2-derived persistent insomnia was associated with significantly lower HRQOL compared to never insomnia in all scales. Additionally, ICSD2-derived persistent insomnia as compared to ICSD2-derived intermittent insomnia was associated with significantly worse total, physical, emotional, social and psychosocial scale scores (Fig. 3).

Both intermittent and persistent insomnia were both individually associated with increased odds of reporting a new chronic medical condition at phase 3 that was not present at phase 1 (Table 5). This effect was seen even after adjustment for age, gender, ethnicity, family income, parent education and presence of OSA (Table 5). The most common new medical condition was asthma; other conditions included allergic rhinitis, cancer, diabetes mellitus, thyroid problems and attention deficit hyperactivity disorder. No significant association between insomnia and a specific medical condition was seen. For ICSD2-derived insomnia, persistent insomnia was associated with an increased risk of starting a new medication at phase 3, as well as specifically starting a new psychiatric medication (Table 5). This effect was still seen after adjustment for age, gender, ethnicity, family income, parent education and presence of OSA. Psychiatric medications reported included stimulants (n = 7), anti-depressants (n = 7) and anti-psychotics (n = 4). The use of sedatives was reported in 2 children, both using clonazepam.

We also examined if baseline insomnia (regardless of future persistence) was predictive of either medical or HRQOL outcomes. Baseline insomnia was predictive of increased risk of reporting a new medical condition at phase 3 for both definitions of insomnia. This was present in univariate (insomnia defined by nighttime symptoms alone: OR 4.1 (1.9–9.1); insomnia derived from ICSD2-criteria: OR 2.7 (1.2–6.5), and multivariate analysis (insomnia defined by nighttime symptoms alone: OR 4.5 (2.0–10.1); insomnia derived from ICSD2-criteria: OR 2.7 (1.1–6.8). No association was seen between baseline insomnia and HRQOL, starting a new medication or starting a new psychiatric medication at phase 3.

Sensitivity analysis revealed that using different sleep duration minimums, including 7 hours, 8 hours, or requiring only 8 hours in place of 9 hours sleep for adolescents did not tangibly change the results for HRQOL and health outcomes. We also evaluated using the ICSD2-derived model with the time in bed requirement omitted. Similar outcomes results were obtained in comparison to the original ICSD2-derived model (see supplemental Fig. 2 and Table 2). Comparable analysis did not show material differences in outcomes for parent vs. child-report at phase 2.

## Discussion

To our knowledge, this is the first community-based longitudinal cohort study that evaluated the relationship between insomnia and HRQOL in children, independent of sleep-disordered breathing and other confounders. Prior studies used a cross-sectional design^39^, or have evaluated the association of insomnia and HRQOL of life in a selected population, such as children with chronic pain^40^ or children with cerebral palsy^41^. In this community-based cohort, insomnia was relatively common in school-going children, and the incidence of ICSD2-derived insomnia was consistent with prior reports^4^,^5^. We found a total prevalence of 30% of ICSD2-derived insomnia in children with a rate of persistence similar to a previous report in Hong Kong Chinese children^5^. Our study shows that insomnia is a common problem in U.S. children and adolescents and that insomnia is associated with incident health-related problems.

Our results show that insomnia in adolescents is associated with worse HRQOL, and that persistence of insomnia is associated with worsened HRQOL. This effect was seen in both a less strict nocturnal symptom based model of insomnia, as well as a stricter ICSD2-derived model of insomnia. In both models of insomnia, a stronger negative association with insomnia was seen in psychosocial aspects of HRQOL, although an association with physical health was also present. These associations were noted even after adjustment for socioeconomic factors, the use of psychiatric medications and OSA. It has been previously suggested that there is only a transient state association between insomnia and poor mental health outcomes in adolescence^5^. Our results show that increased persistence of insomnia is associated with worse psychosocial HRQOL, which may indicate the presence of a trait effect of insomnia on mental health outcomes.

Our results also show that insomnia may be associated with an increased risk of developing a chronic medical condition. These results are consistent with prior studies demonstrating an association between insomnia and chronic disease, such as upper airway disease, in children^5^. There is evidence that this association between insomnia and chronic disease is likely bidirectional^14^,^42^.

This study does have several limitations. Nocturnal insomnia symptoms were reported as present or absent, therefore, frequency information (how many times per week) was not available. This limitation has been present in prior research as well^20^. We used a threshold of frequently for daytime symptoms to attempt to mitigate this limitation. Additionally, daytime symptom questions did not specifically address whether the reported symptom was directly related to insomnia, as the questionnaire was designed to detect symptoms of insomnia, but not to diagnose insomnia per the ICSD2 definition of insomnia. Similarly, the cohort was not specifically recruited to study insomnia. Mood and concentration related symptoms, while included as daytime symptoms in the ICSD2 definition of insomnia, were not included in the case selection criteria, as these domains were not included in the original sleep questionnaire, and are assessed by the PedsQL; therefore inclusion would bias our outcome measurements. There was a high dropout rate among subjects (38% attrition after both phase 1 and phase 2), likely secondary to the long duration of the study and rigorous nature of study procedures at phase 1 and 2. The dropout rate from phase 1 to 2 was consistent with a similar longitudinal cohort study, the Penn State Child cohort^43^. Significantly, there were no significant differences seen in insomnia symptoms between the study sample and the attrition group. Additionally, adjustment was performed for the variables–ethnicity and family income–that were significantly different between the participants who remained in the study when compared to those who were lost to follow-up. The cohort was initially recruited to determine the prevalence of sleep-disordered breathing in a community cohort of children, and not specifically insomnia. However, the presence of OSA was not different between the children with and without insomnia, suggesting this may not have influenced our results. The presence of OSA was assessed via polysomnography at phase 1 and 2, but by self-report at phase 3. This may have resulted in a lower prevalence of OSA at phase 3 than if polysomnography had been used to assess for OSA. To attempt to account for this, we performed sensitivity analysis using history of OSA at any time point, in place of OSA reported at phase 3, but results were not materially different from those reported. The prevalence of insomnia based on nocturnal symptoms alone was higher than previously reported. This is likely due to the requirement of a positive response to only a single question to meet the case definition. Additionally, children were assessed at 3 time points, while other studies have generally only assessed symptoms at 1 or 2 time points. This likely resulted in a higher prevalence of insomnia, as there were more opportunities to be classified as having insomnia. Our stricter, ICSD2-derived criteria resulted in a prevalence more consistent with previous results. Only Hispanic and Caucasian children were included in the study, and the results may not be generalizable to other racial groups or ethnicities. Nevertheless despite these limitations, our study has multiple strengths. The use of a nine hour minimum sleep duration for ICSD2-derived insomnia likely minimized the impact of short sleep duration as a potential cause of worsened HRQOL. However, this would not have identified short sleep duration with sufficient time in bed but environmental disruptions impairing sleep. We were able to adjust for several important covariates that influence HRQOL, including socioeconomic factors, the presence of obstructive sleep apnea and the use of psychiatric medications. Additionally, we were able to follow children over a time period of 7 years to obtain longitudinal data spanning from childhood to adolescence.

## Conclusion

Our study demonstrates that insomnia is common in children, and is associated with worse HRQOL and poor health outcomes. Future research is needed to determine the public health implications of childhood insomnia. Additional investigation is also needed to evaluate the effects of insomnia interventions on HRQOL and incident psychiatric and medical conditions in children and adolescents.

## Additional Information

**How to cite this article**: Combs, D. *et al*. Insomnia, Health-Related Quality of Life and Health Outcomes in Children: A Seven Year Longitudinal Cohort. *Sci. Rep.* **6**, 27921; doi: 10.1038/srep27921 (2016).

## Supplementary Material

Supplementary Information

## Figures and Tables

**Table 1:** Characteristics of participants who remained in the study in comparison to those lost to follow-up.

	Attrition Group (n = 309)	Cohort in this report (n = 194)	p
**Gender**			0.93
Male	154 (50%)	98(51%)	
Female	155 (50%)	96 (49%)	
Ethnicity			0.002
Caucasian	166 (54%)	131 (68%)	
Hispanic	143 (46%)	63 (32%)	
Family Income			0.03
<$40,000	68 (47%)	68 (35%)	
≥$40,000	76 (53%)	126 (65%)	
Parent Education			0.1
<4 years college	95 (61%)	102 (53%)	
≥4 years college	61(39%)	92 (47%)	
Age‡	9.0 (±1.6)	8.7 (± 1.6)	0.1
Obstructive Sleep Apnea†	58 (19%)	31 (16%)	0.5
Insomnia§	80 (26%)	52 (27%)	0.8
Insomnia (ICSD2-derived)**	32 (11%)	20 (10%)	1

^*^All measurements were obtained in phase 1.

^‡^Age is reported as mean ± standard deviation.

^†^Obstructive sleep apnea was defined as an apnea-hypopnea index ≥ 1.5 on unattended polysomnography. OSA: obstructive sleep apnea.

^§^Insomnia requiring only the subjective complaint of problems falling asleep, staying asleep or waking too early in the morning.

^**^International Classification of Sleep Disorders (ICSD2) derived insomnia includes subjective complaint of problems falling asleep, staying asleep or waking too early in the morning with the additional requirements of ≥ 9 hours of time in bed as well as daytime symptom including learning problems, daytime sleepiness, or concern about not getting enough sleep.

**Table 2:** Demographics of participants at various phases of the TuCASA cohort.

	Phase 1	Phase 2	Phase 3
Gender			
Male	98 (51%)	98 (51%)	98 (51%)
Female	96 (49%)	96 (49%)	96 (49%)
Ethnicity			
Caucasian	131 (67%)	131 (67%)	131 (67%)
Hispanic	63 (33%)	63 (33%)	63 (33%)
Age‡	8.7 (± 1.6)	13.1 (± 1.6)	15 (( ± 1.8)
Obstructive sleep apnea(OSA)*	31 (16%)	14 (7%)	
Reported diagnosis of OSA			4 (2%)
Difficulty falling asleep	34 (18%)	65 (34%)	74 (38%)
Difficulty staying asleep	16 (8%)	24 (12%)	30 (16%)
Early morning awakening	24 (12%)	54 (28%)	49 (25%)
Time in bed ≥ 9 hours	187 (96%)	115 (59%)	54 (28%)
Daytime sleepiness	15 (8%)	48 (25%)	61 (31%)
Insufficient sleep	21 (11%)	55 (28%)	70 (36%)
Learning problems	7 (4%)	10 (5%)	13 (7%)
Insomnia (nighttime symptoms alone)§	52 (27%)	104 (54%)	98 (51%)
Insomnia with daytime sleepiness	4 (2%)	34 (18%)	41 (21%)
Insomnia with daytime impairment**	21 (11%)	37 (19%)	67 (35%)
Insomnia (ICSD2-derived)***	20 (10%)	36 (19%)	16 (8%)

^‡^Age is reported as mean ± standard deviation.

^*^In phase 1 and 2, polysomnography was performed, and an apnea-hypopnea index ≥ 1.5 determined obstructive sleep apnea. For phase 3, obstructive sleep apnea was assessed by asking participants if they had been diagnosed with obstructive sleep apnea.

^§^Insomnia requiring only the subjective complaint of problems falling asleep, staying asleep or waking too early in the morning.

^**^Insomnia including nighttime symptoms plus daytime symptoms including learning problems, daytime sleepiness, or concern about not getting enough sleep.

^***^International Classification of Sleep Disorders (ICSD2) derived insomnia includes subjective complaint of problems falling asleep, staying asleep or waking too early in the morning with the additional requirements of ≥ 9 hours of time in bed as well as daytime symptoms including learning problems, daytime sleepiness, or concern about not getting enough sleep.

**Table 3:** Demographics of participants with ICSD2-derived insomnia in the TuCASA study.

	No insomnia	Intermittent Insomnia	Persistent insomnia	p
Gender				0.4
Male	65 (48%)	28 (54%)	5 (71%)	
Female	70 (52%)	24 (46%)	2 (29%)	
Ethnicity				0.2
Caucasian	95 (70%)	30 (58%)	6 (86%)	
Hispanic	40 (30%)	22 (42%)	1 (14%)	
Age‡	15 (±1.7)	15 (±1.7)	13 (±1.0)	0.001
Parent education level				0.3
< 4 years of college	66 (49%)	31 (60%)	5 (71%)	
≥ 4 years of college	69 (51%)	21 (40%)	2 (29%)	
Family income				0.4
<$40,000	45 (33%)	19 (37%)	4 (57%)	
≥$40,000	90 (67%)	33 (63%)	3 (43%)	
Current use of psychiatric medication				0.007
No	128 (95%)	47 (90%)	4 (57%)	
Yes	7 (5%)	5 (10%)	3 (43%)	
Obstructive sleep apnea				0.3
Absent	133 (99%)	50 (96%)	7 (100%)	
Present	2 (1%)	2 (4%)	0 (0%)	
New Medication				0.03
No	117 (87%)	39 (75%)	4 (57%)	
Yes	18 (13%)	13 (25%)	3 (43%)	
New Psychiatric Medication				0.01
No	128 (95%)	48 (92%)	4 (57%)	
Yes	7 (5%)	4 (8%)	3 (43%)	
New medical condition				0.15
No	117 (87%)	39 (58%)	6 (86%)	
Yes	18 (13%)	13 (42%)	1 (14%)	

The use of psychiatric medication, starting a new medication, starting a new psychiatric medication and age were significantly different between groups. All data is from phase 3, except socioeconomic data, which was collected at phase 2.

^‡^Age is reported as mean ± standard deviation.

**Table 4:** Regression of insomnia and health-related quality of life.

	Peds QL total		Physical		Emotional		Social		School		Psychosocial	
	B (SE)	p	B (SE)	p	B (SE)	p	B (SE)	p	B (SE)	p	B (SE)	p
Age	−0.8 (0.6)	0.1	−0.8 (0.5)	0.1	−1.5 (0.9)	0.07	0.6 (0.7)	0.3	−1.6 (0.8)	0.03	−0.8 (0.6)	0.2
Female gender	−1.2 (1.9)	0.53	−3.1 (1.8)	0.1	−4.8 (3.0)	0.1	3.4 (2.4)	0.2	0.6 (2.7)	0.8	−0.3 (2.3)	0.9
Caucasian	−1.4 (2.1)	0.5	−2.7 (1.9)	0.2	0.9 (3.2)	0.8	−4.4 (2.5)	0.1	1.5 (2.9)	0.6	−0.7 (2.4)	0.8
Income <$40,000	−0.9 (2.0)	0.7	−2.1 (1.9)	0.3	1.9 (3.1)	0.5	−0.5 (2.5)	0.9	−2.3 (2.8)	0.4	−0.3 (2.4)	0.9
<4 years college	−2.2 (1.9)	0.3	−0.7 (1.8)	0.7	−4.1 (3.0)	0.2	−3.5 (2.4)	0.1	−3.7 (2.7)	0.2	−3.8 (2.2)	0.1
Use of psychiatric medication	−17.1 (3.4)	<0.001	−5.7 (3.3)	0.1	−20.4 (5.4)	<0.001	−21.7 (4.2)	<0.001	−27.6 (4.7)	<0.001	−23.2 (3.9)	<0.001
Obstructive sleep apnea	−4.3 (6.8)	0.5	−6.0 (6.3)	0.3	−5.9 (10.5)	0.6	−1.4 (8.4)	0.9	−2.8 (9.5)	0.8	−3.4 (8.0)	0.7
Intermittent insomnia	−9.9 (2.1)	<0.001	−8.0 (2.3)	<0.001	−14.3 (3.2)	<0.001	−10.2 (2.7)	<0.001	−8.4 (3.1)	0.007	−11.0 (2.4)	<0.001
Persistent insomnia	−11.7 (2.4)	<0.001	−7.1 (2.3)	0.002	−20.8 (3.7)	<0.001	−11.9 (3.1)	<0.001	−9.6 (3.5)	0.007	−14.1 (2.8)	<0.001
Intermittent ICSD2-derived insomnia	−19.0 (5.1)	<0.001	−13.9 (4.9)	0.005	−21.0 (8.1)	0.01	−34.2 (6.1)	<0.001	−10.2 (7.4)	0.17	−21.8 (5.9)	<0.001
Persistent ICSD2-derived insomnia	−24.5 (4.9)	<0.001	−16.5 (4.7)	0.001	−29.0 (7.8)	<0.001	−38.9 (5.9)	<0.001	−18.5 (7.1)	0.01	−28.9 (5.7)	<0.001

Insomnia was defined as presence of at least one of the following symptoms: trouble falling asleep, staying asleep, or waking up too early in the morning. ICSD2-derived insomnia was determined by the International Classification of Sleep Disorders (ICSD2) criteria. ICSD2-derived insomnia required the presence of at least one of the following symptoms of trouble falling asleep, staying asleep, or waking up too early in the morning, in addition to reporting adequate time in bed (≥9 hours) and the presence of at least one of the following daytime symptoms: learning problems, daytime sleepiness, or concern about not getting enough sleep.

**Table 5:** Logistic regression of insomnia and health problems.

	New medical condition		New medications		New psychiatric medications	
	OR (95% CI)	P value	OR (95% CI)	P value	OR (95% CI)	P value
Unadjusted						
Intermittent insomnia	5.9 (1.3–26.7)	0.04	1.8 (0.7–4.9)	0.3	1.8 (0.3–9.2)	0.5
Persistent insomnia	8.0 (2.3–27.7)	0.001	1.4 (0.5–4.1)	0.5	2.3 (0.4–11.9)	0.3
Intermittent ICSD2-derived insomnia	2.2 (1.0–4.8)	0.06	2.2 (1.0–4.8)	0.06	1.5 (0.4–5.4)	0.5
Persistent ICSD2-derived insomnia	1.1 (0.1–9.5)	0.4	4.9 (1.0–23.6)	0.049	13.7 (2.6–73.5)	0.002
Adjusted						
Intermittent insomnia	6.9 (1.4–34.0)	0.02	1.8 (0.6–5.4)	0.3	2.1 (0.3–13.8)	0.4
Persistent insomnia	12.4 (1.5–102.1)	0.02	1.6 (0.5–5.1)	0.4	2.2 (0.3–14.0)	0.2
Intermittent ICSD2-derived insomnia	2.2 (1.0–5.0)	0.06	2.4 (1.0–5.5)	0.05	1.5 (0.4–5.7)	0.6
Persistent ICSD2-derived insomnia	1.1 (0.1–11.4)	0.9	11.1 (1.5–79.5)	0.02	46.3 (3.5–609.4)	0.004

Odds ratios were adjusted for presence of obstructive sleep apnea, age, gender, ethnicity, family income and parent education. Insomnia was defined as presence of at least one of the following symptoms: trouble falling asleep, staying asleep, or waking up too early in the morning. ICSD2-derived insomnia was determined by the International Classification of Sleep Disorders (ICSD2) criteria. ICSD2-derived insomnia required the presence of at least one of the following symptoms: trouble falling asleep, staying asleep, or waking up too early in the morning, in addition to reporting adequate time in bed (≥9 hours) and the presence of at least one of the following daytime symptoms: learning problems, daytime sleepiness, or concern about not getting enough sleep. OR: Odds ratio, CI: confidence interval.
