# Supplementary material for: Insomnia, Health-Related Quality of Life and Health Outcomes in Children: A Seven Year Longitudinal Cohort
Source: Sci Rep. 2016 Jun 13;6:27921. doi: 10.1038/srep27921 (PMC4904740; doi:10.1038/srep27921)
Supplement: Supplementary Information [file srep27921-s1.pdf]

Insomnia, Health-Related Quality of Life and Health Outcomes in Children: A Seven Year Longitudinal Cohort

Daniel Combs, James L. Goodwin, Stuart F. Quan, Wayne J. Morgan, Safal Shetty, Sairam Parthasarathy

Supplemental Information:

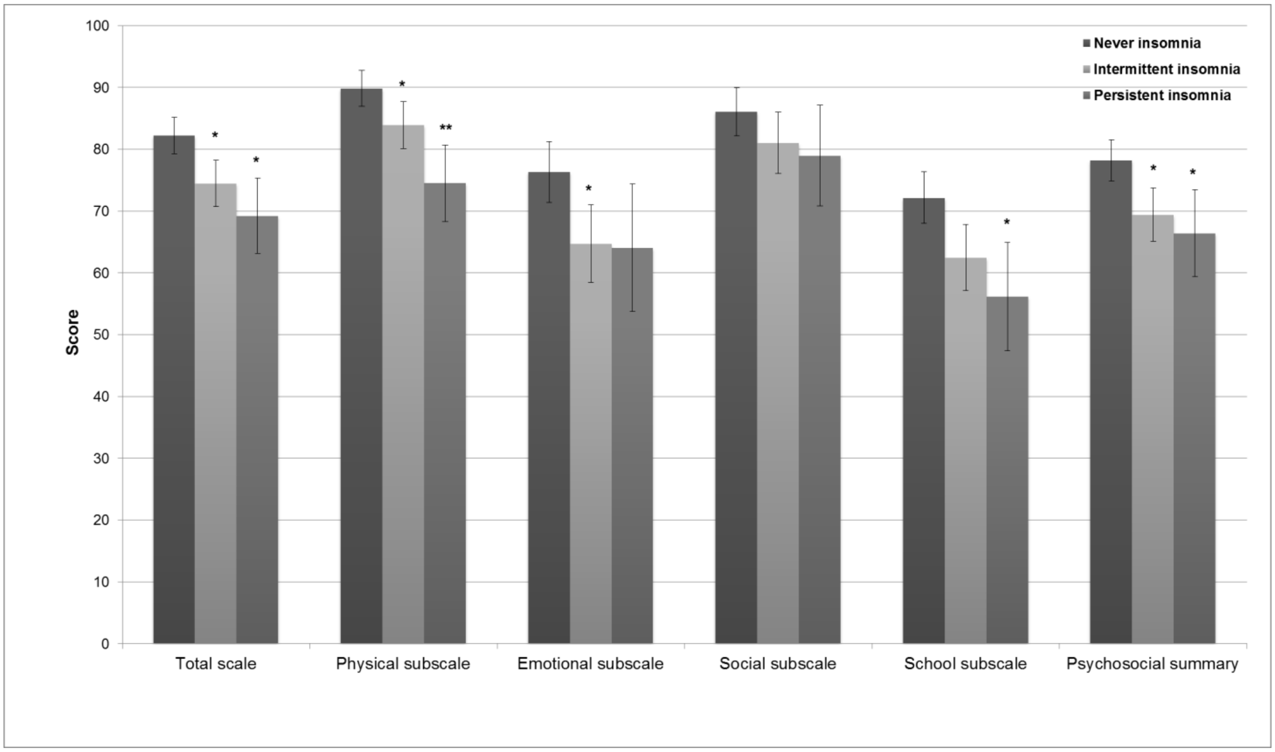

Supplemental Figure 1. PedsQL scores in subjects with insomnia based on presence of nocturnal symptoms and daytime sleepiness, without a minimum time in bed requirement. The presence of insomnia is associated with decreased HRQOL across all domains except the physical and school scale. Persistent insomnia is associated with further worsening of overall, social and psychosocial HRQOL. *Significantly different ($p<.05$) from no insomnia, **significantly different from intermittent insomnia and no insomnia. Insomnia required the presence of at least one of the following symptoms of trouble falling asleep, staying asleep, or waking up too early in the morning in addition to the presence of daytime sleepiness. In this model of insomnia, 139 subjects (68%) never had insomnia, 55 (28%) had intermittent insomnia and 12 (6%) had persistent insomnia.

Supplemental Table 1: Logistic regression of insomnia (nocturnal symptoms plus daytime sleepiness, no time in bed requirement) and health problems.

	New medical condition		New medications		New psychiatric medications	
	**OR (95% CI)**	**P value**	**OR (95% CI)**	**P value**	**OR (95% CI)**	**P value**
**Unadjusted**						
Intermittent insomnia	2.5 (1.2-5.5)	0.02	2.3 (1.05-5.0)	0.04	2.7 (0.8-8.8)	0.10
Persistent insomnia	*	*	1.3 (0.3-6.6)	0.7	4.4 (0.8-24.9)	0.09
**Adjusted**						
Intermittent insomnia	2.7 (1.2-6.1)	0.02	2.4 (1.1-5.4)	0.04	2.9 (0.8-10.0)	0.10
Persistent insomnia	*	*	1.4 (0.3-7.5)	0.07	4.7 (0.7-31.6)	0.11

Odds ratios were adjusted for presence of obstructive sleep apnea, age, gender, ethnicity, family income and parent education. Insomnia required the presence of at least one of the following symptoms of trouble falling asleep, staying asleep, or waking up too early in the morning in addition to the presence of daytime sleepiness. *Due to small sample size, a regression model could not be created.

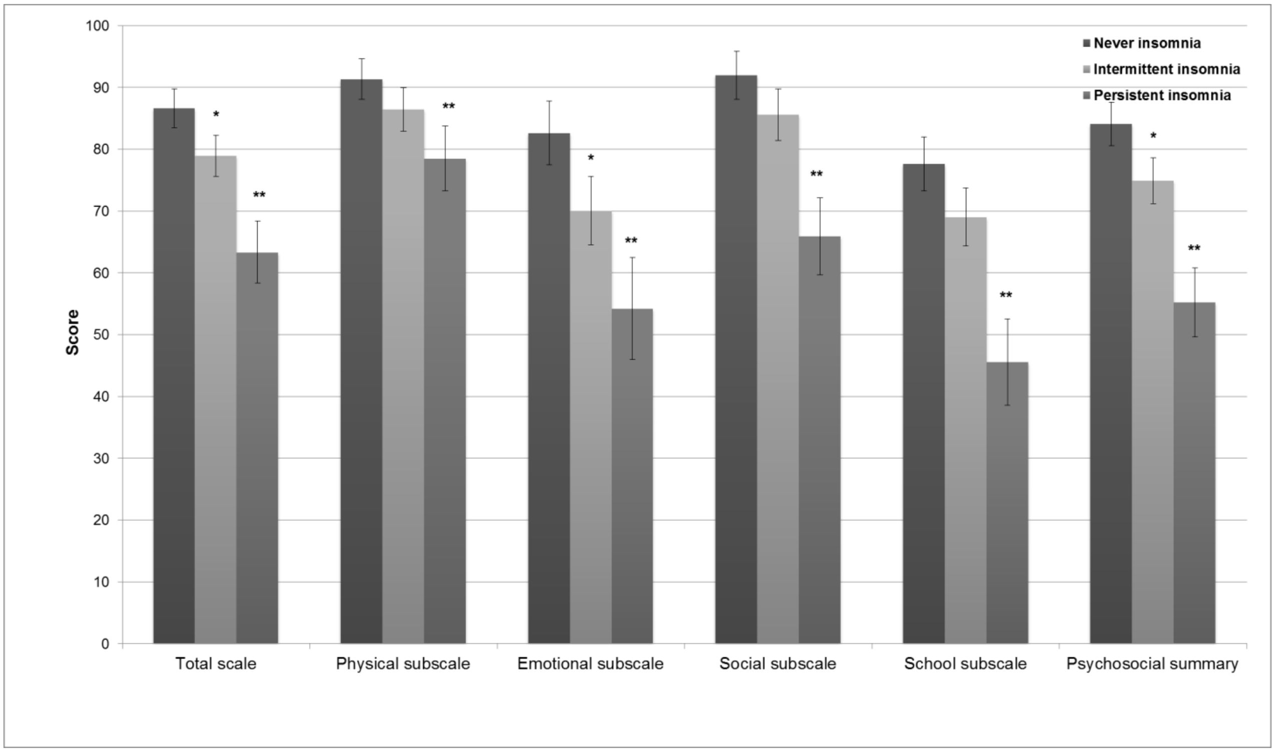

Supplemental Figure 2. PedsQL scores in subjects with insomnia based on presence of nocturnal and daytime symptoms, but no time in bed requirement. The presence of insomnia is associated with decreased HRQOL across all domains except the physical and school scale. Persistent insomnia is associated with further worsening of overall, social and psychosocial HRQOL. *Significantly different ($p<.05$) from no insomnia, **significantly different from intermittent insomnia and no insomnia. Insomnia required the presence of at least one of the following symptoms of trouble falling asleep, staying asleep, or waking up too early in the morning in addition to the presence of at least one of the following daytime symptoms -- learning problems, daytime sleepiness, or concern about not getting enough sleep. In this model of insomnia, 103 subjects (53%) never had insomnia, 70 (36%) had intermittent insomnia and 21 (11%) had persistent insomnia.

Supplemental Table 2: Logistic regression of insomnia (nocturnal and daytime symptoms, no time in bed requirement) and health problems.

	**New medical condition**		**New medications**		**New psychiatric medications**	
	**OR (95% CI)**	**P value**	**OR (95% CI)**	**P value**	**OR (95% CI)**	**P value**
**<u>Unadjusted</u>**						
Intermittent insomnia	0.7 (0.3-1.6)	0.41	0.5 (0.2-1.2)	0.14	0.5 (0.1-2.0)	0.35
Persistent insomnia	3.5 (1.2-10.2)	0.02	4.7 (1.6-13.6)	0.005	7.7 (1.9-32.0)	0.005
**<u>Adjusted</u>**						
Intermittent insomnia	0.7 (0.3-1.7)	0.43	0.5 (0.2-1.2)	0.13	0.5 (0.1-2.2)	0.36
Persistent insomnia	3.9 (1.2-12.8)	0.03	5.7 (1.7-19.8)	0.006	8.8 (1.7-46.2)	0.01

Odds ratios were adjusted for presence of obstructive sleep apnea, age, gender, ethnicity, family income and parent education. Insomnia required the presence of at least one of the following symptoms of trouble falling asleep, staying asleep, or waking up too early in the morning in addition to the presence of at least one of the following daytime symptoms -- learning problems, daytime sleepiness, or concern about not getting enough sleep.

Supplemental Table 3: Polysomnography results of children with ICSD2-derived insomnia

	No insomnia	Intermittent Insomnia	Persistent insomnia	p
**Phase 1**				
**Sleep latency (minutes)**	15 (12-17)	17 (9-24)	14 (1-28)	0.83
**Sleep efficiency (%)**	90 (89-91)	91 (89-92)	90 (89-91)	0.67
**Total sleep time**	8:04 (7:49-8:20)	8:10 (7:49-8:31)	7:56 (6:05-9:48)	0.89
**Stage 1 sleep (%)**	4.5 (3.9-5.1)	3.9 (3.2-4.5)	2.4 (0.8-4.1)	0.13
**Stage 2 sleep (%)**	53.2 (51.5-54.8)	56.4 (52.5-60.2)	58.2 (39.7-76.7)	0.15
**Stage 3 sleep (%)**	21.9 (20.6-23.2)	20.2 (17.8-22.5)	19.8 (7.4-32.2)	0.37
**REM sleep (%)**	20.4 (19.4-21.4)	20.2 (17.8-22.5)	19.6 (10.8-28.4	0.75
**Arousal Index (arousals/hour)**	3.6 (3.4-3.8)	3.2 (3.0-3.5)	3.9 (3.1-4.7)	0.14
**Phase 2**				
**Sleep latency (minutes)**	30 (25-37)	25 (17-32)	39 (16-61)	0.42
**Sleep efficiency (%)**	87 (86-88)	88 (86-90)	82 (74-90)	0.12
**Total sleep time**	7:46 (7:35-7:56)	7:41 (7:21-8:02)	7:52 (6:57-9:47)	0.88
**Stage 1 sleep (%)**	3.9 (3.5-4.2)	3.9 (3.2-4.6)	6.0 (1.6-10.4)	0.053
**Stage 2 sleep (%)**	54.4 (53.3-55.6)	55.8 (53.9-57.7)	51 (42.0-60.2)	0.2
**Stage 3 sleep (%)**	3.9 (3.6-4.2)	3.9 (3.2-4.7)	3.8 (1.2-6.5)	0.99
**REM sleep (%)**	22.7 (22.0-23.5)	21.7 (20.3-23.1)	22.5 (21.8-23.1)	0.43
**Arousal Index (arousals/hour)**	6.1 (5.7-6.5)	6.6 (5.7-7.5)	6.7 (3.9-9.6)	0.47

Data is reported as mean (95% confidence interval). ICSD2-derived insomnia was determined by the International Classification of Sleep Disorders (ICSD2) criteria. ICSD2-derived insomnia required the presence of at least one of the following symptoms of trouble falling asleep, staying asleep, or waking up too early in the morning, in addition to reporting adequate time in bed (≥ 9 hours) and the presence of at least one of the following daytime symptoms: learning problems, daytime sleepiness, or concern about not getting enough sleep. Similar results were seen using the night-time symptoms only definition of insomnia. No sleep architecture variables were significantly different between groups in either model of insomnia.

The questions related to insomnia from the Tucson Children's Assessment of Sleep Apnea (TuCASA) are provided below:

Sleep symptoms:

Has this child ever been troubled by any of the following sleep problems:

1. Trouble falling to asleep?
2. Trouble staying asleep?
3. Waking up too early and not being able to get back to sleep?

Answer choices included "Yes, still has this problem; Yes, but no longer has this problem; No, does not have the problem." An answer of "Yes, still has this problem" was considered positive, all others negative.

Daytime symptoms:

1. Is your child sleepy during the daytime?
2. Does your child have learning problems?
3. Not enough sleep?

Answer choices for question 1 and 2 included "Don't Know, Never, Rarely, Occasionally, Frequently, Almost always." An answer of Frequently or Almost always was considered a positive response.

Answer choices for question 3 included “Yes, still has this problem; Yes, but no longer has this problem; No, does not have the problem.” An answer of “Yes, still has this problem” was considered positive, all others negative.

Tucson Children Assessment of Sleep Apnea<br>*SLEEP HABITS QUESTIONNAIRE*	**TuCASA ID#:** ________________<br>**Today's Date:** _____/_____/______<br>mon day year

We would like to ask you some questions regarding your child's sleep and health. Please do your best to answer these questions regarding your child's sleep over THE LAST TWO MONTHS. The answers will help us to understand the results of tonight's test. Please complete as thoroughly as possible and to the best of your knowledge.

1. At what time does this child usually go to bed on school nights? _______:_______ am/pm

2. At what time does this child usually go to bed on non-school nights? _______:_______ am/pm

3. How long does it usually take this child to fall asleep after going to bed? ______hrs______min

4. At what time does this child awaken on school days? _______:_______ am/pm

5. At what time does this child awaken on non-school days? _______:_______ am/pm

6. How many hours of sleep does this child usually get on school nights? ______hrs______min

7. How many hours of sleep does this child usually get on non-school nights? ______hrs______min

8. During a usual week, how many times does this child take a nap for 5 minutes or more? (Write in "0" if he/she does not take any naps.) ________#

9. Has this child ever been troubled by any of the following sleep problems?(Please check the appropriate box)

	Yes, still has the problem	Yes, but no longer has problem	No does not have the problem
a. Trouble falling asleep?	☐ 1	☐ 2	☐ 3
b. Trouble staying asleep?	☐ 1	☐ 2	☐ 3
c. Too much sleep?	☐ 1	☐ 2	☐ 3
d. Not enough sleep?	☐ 1	☐ 2	☐ 3
e. Waking up too early and not being able to get back to sleep?	☐ 1	☐ 2	☐ 3
f. Falling asleep during the day?	☐ 1	☐ 2	☐ 3
g. Wake up screaming during the night?	☐ 1	☐ 2	☐ 3
h. Banging his or her head or rocking his or her body when going to sleep?	☐ 1	☐ 2	☐ 3

10. Does this child wet the bed at night?

- ☐ 1 Never
- ☐ 2 Less than 5 times per month
- ☐ 3 5-10 times per month
- ☐ 4 More than 10 times per month

11a. How often does this child awaken at night afraid or appearing tearful?

- ☐ 1 Never (If never, go to Question 12)
- ☐ 2 Less than 5 times per month
- ☐ 3 5-10 times per month
- ☐ 4 More than 10 times per month

b. After awakening from one of these episodes, can this child tell you about a dream?

☐ 1 Yes ☐ 2 No

12. Does this child sleepwalk?	☐ 1 Never<br>☐ 2 Less than 3 times per month<br>☐ 3 3-5 times per month<br>☐ 4 More than 5 times per month
13. Does this child talk in his or her sleep?(Talk without being fully awake?)	☐ 1 Never<br>☐ 2 Less than 3 times per month<br>☐ 3 3-5 times per month<br>☐ 4 More than 5 times per month
14. Does this child not seem to listen when spoken to directly?	☐ 1 Yes ☐ 2 No
15. Is this child easily distracted?	☐ 1 Yes ☐ 2 No
16. Is this child easily distracted by extraneous stimuli?	☐ 1 Yes ☐ 2 No
17. Does this child fidget with hands or feet or squirm in his seat?	☐ 1 Yes ☐ 2 No
18. Is this child "on the go" or often act as if "driven by a motor"?	☐ 1 Yes ☐ 2 No
19. Does this child interrupt or intrude on others)eg, butts into conversations or games)?	☐ 1 Yes ☐ 2 No
20. Has this child ever had their tonsils and/or adenoids removed?	☐ 1 Yes ☐ 2 No
21. Is this child currently under the care of a doctor for any medical or physical condition?<br><br>If yes, please specify the condition(s) ______________________	☐ 1 Yes ☐ 2 No

22. How would you assess this child's activity level compared to other children of his or her age?

(Circle your choice)

Much Less		Same		Much More
1	2	3	4	5

23. Does your child have restless sleep?	☐ 1 Yes ☐ 2 No
24. Does your child describe restlessness of the legs when in bed?	☐ 1 Yes ☐ 2 No
25. Does your child have "growing pains" (unexplained leg pains)?	☐ 1 Yes ☐ 2 No
26. Does your child have "growing pains" that are worse in bed?	☐ 1 Yes ☐ 2 No
27. When your child sleeps, have you seen brief kicks of one leg or both legs?	☐ 1 Yes ☐ 2 No

28. When your child sleeps, have you seen repeated kicks or jerks of the legs at regular intervals (i.e., about every 20 to 40 seconds)?

☐ 1 Yes ☐ 2 No

29. Does your child complain of unpleasant sensations such as aching, tickles, crawling or spiders in their legs combined with an urge or need to move their legs?

☐ 1 Yes ☐ 2 No (If no, go to Question 33)

30. If yes to 29, does your child indicate that these feelings occur mainly or only at rest, and do they improve with movement?

☐ 1 Yes ☐ 2 No

31. If yes to 29, does your child indicate that these feelings are worse in the evening or night than in the morning

☐ 1 Yes ☐ 2 No

32. If yes to 29, how often does your child tell you that these feelings occur?

☐ 1 Less than one time per year
☐ 2 At least one time a year, but less than one time per month
☐ 3 One time per month
☐ 4 2-4 times per month
☐ 5 2-3 times per week
☐ 6 4-5 times per week
☐ 7 6-7 times per week

33. Does your child have difficulty with reading and writing?

☐ 1 Yes ☐ 2 No

34. Does your child learn best on a one-to-one basis?

☐ 1 Yes ☐ 2 No

35. How well does your child do in school?

☐ 1 Gets mostly A's or is outstanding
☐ 2 Gets mostly A's and B's or is excellent
☐ 3 Gets mostly B's or is very good
☐ 4 Gets mostly B's and C's or is good
☐ 5 Gets mostly C's or is satisfactory/average
☐ 6 Gets mostly grades worse than C's or is below average
☐ 7 Doesn't get grades
☐ 8 Doesn't know

36. Did this child's biological father ever have any of the following?

	Yes	No	Don't Know
a. Loud snoring?	☐ 1	☐ 2	☐ 3
b. Sleep apnea (a condition in which breathing stops briefly during sleep	☐ 1	☐ 2	☐ 3
c. Obesity?	☐ 1	☐ 2	☐ 3
d. Insomnia?	☐ 1	☐ 2	☐ 3
e. Excessive daytime sleepiness?	☐ 1	☐ 2	☐ 3
f. Other sleep problem?	☐ 1	☐ 2	☐ 3

Please specify:

37. Did this child's biological mother ever have any of the following?

	Yes	No	Don't Know
a. Loud snoring?	☐ 1	☐ 2	☐ 3
b. Sleep apnea (a condition in which breathing stops briefly during sleep	☐ 1	☐ 2	☐ 3
c. Obesity?	☐ 1	☐ 2	☐ 3
d. Insomnia?	☐ 1	☐ 2	☐ 3
e. Excessive daytime sleepiness?	☐ 1	☐ 2	☐ 3
f. Other sleep problem?	☐ 1	☐ 2	☐ 3

Please specify:

38. Does anyone in this child's household smoke in the house? ☐ 1 Yes ☐ 2 No

If "Yes", please indicate all the individuals in the household who smoke:

	Yes	No	Don't Know
a. Father	☐ 1	☐ 2	☐ 3
b. Mother	☐ 1	☐ 2	☐ 3
c. Step-mother	☐ 1	☐ 2	☐ 3
d. Step-father	☐ 1	☐ 2	☐ 3
e. Brother	☐ 1	☐ 2	☐ 3
f. Sister	☐ 1	☐ 2	☐ 3
g. Other	☐ 1	☐ 2	☐ 3

39. Does your child drink caffeinated beverages on a typical day (cola, tea coffee, etc)? ☐ 1 Yes ☐ 2 No

40. If yes to 39, how many cups or cans per day? ________cups/cans

41. What medication(s) is(are) this child currently taking?

42. What is the highest grade or year of school you have ever completed, including trade or vocational school, college, and graduate or professional school?

☐ 0 Grades 1-12 (record number; use "0" for none) _____ _____

☐ 1 GED

Vocational School:

☐ 2 1 year

☐ 3 2 years

☐ 4 3 years

College:

☐ 5 1 year

☐ 6 2 years

☐ 7 3 years

☐ 8 4 years

☐ 9 Graduate or Professional

43. Which best describes the kind of work you have done most of your life?

- ☐ 1 Professional/Technical/Managerial/Administrative
- ☐ 2 Sales/Clerical Service
- ☐ 3 Craftsman/Machine Operator/Laborer
- ☐ 4 Farming/Forestry
- ☐ 5 Housewife
- ☐ 6 Other

(specify) ______________________________________

44. Which of these income groups represents your total combined family income, before taxes for the past 12 months? Include income from all sources such as wages, salaries, social security or retirement benefits, help from relatives, rent from property, and so forth.

Yearly	Monthly	
Less than $5,000	Less than $416	☐ 1
$5,000 to $9,999	$417 to $833	☐ 2
$10,000 to $14,999	$834 to $1,250	☐ 3
$15,000 to $19,999	$1,251 to $1,666	☐ 4
$20,000 to $24,999	$1,667 to $2,083	☐ 5
$25,000 to $29,999	$2,084 to $2,500	☐ 6
$30,000 to $34,999	$2,501 to $2,917	☐ 7
$35,000 to $39,999	$2,918 to $3,333	☐ 8
$40,000 to $44,999	$3,334 to $3,750	☐ 9
$45,000 to $49,999	$3,751 to $4,166	☐ 10
More than $50,000	More than $4,166	☐ 11

45. Who completed this questionnaire?

- ☐ 1 Self-completed by child
- ☐ 2 Father
- ☐ 3 Mother
- ☐ 4 Step-mother
- ☐ 5 Step-father
- ☐ 6 Other
  Please specify:

In case we lose contact with you in the future, we'd appreciate having the name and phone number of someone who would know how to reach you (preferably someone who does not live with you). Thanks!

CONTACT PERSON: ____

RELATIONSHIP TO YOU: ____

PHONE: ______________________

For Office use only	
Date Reviewed: _____/_____/______ (mon day year)	By Tech ID#_________

Thank you very much for participating in the TuCASA Study!
